# Three-Dimensional Organization of Telomeres: An Emerging Prognostic Biomarker in Multiple Myeloma

**DOI:** 10.3390/cells14231890

**Published:** 2025-11-28

**Authors:** Yulia Shifrin, Sabine Mai

**Affiliations:** 1 Telo Genomics Corp., Toronto, ON M5V 3B1, Canada; 2 Department of Physiology & Pathophysiology, Rady Faculty of Health Sciences, University of Manitoba, Winnipeg, MB R3T 2N2, Canada; sabine.mai@umanitoba.ca

**Keywords:** multiple myeloma, minimal residual disease, genome instability, telomeric dysfunction, 3D telomere profiling, nuclear architecture, prognostic biomarkers

## Abstract

**Highlights:**

**What are the main findings?**

- Telomere parameters, such as telomere number, intensity, aggregates, and spatial organization, were identified as prognostic biomarkers in multiple myeloma (MM).
- Changes in the three-dimensional telomeric organization may predict risk of relapse in newly diagnosed MM and risk of progression in smoldering MM, with similar profiles in bone marrow and peripheral blood cells.

**What are the implications of the main findings?**

- 3D telomere profiling may enable minimally invasive monitoring of MM status and measurable residual disease using liquid biopsies.
- This approach potentially addresses an unmet clinical need by improving risk stratification and guiding personalized treatment strategies in multiple myeloma.

**Abstract:**

A crucial role of genome instability and telomeric dysfunction was demonstrated in multiple cancers, including multiple myeloma (MM). MM accounts for approximately 10% of all hematologic malignancies and includes asymptomatic pre-malignant monoclonal gammopathy of undetermined significance (MGUS) and smoldering multiple myeloma (SMM). Due to the highly heterogeneous nature of the disease, there is an ongoing need for precise risk stratification and subsequent development of risk-adapted treatment strategies at every stage of disease and during disease progression. Telomere numbers, intensity, aggregates, and spatial arrangement within the nucleus were identified as prognostic biomarkers. Recent studies demonstrated that the three-dimensional (3D) analysis of key telomeric parameters is a reliable marker of the high risk of relapse in newly diagnosed MM (NDMM) patients and can predict the risk of progression of SMM patients. Telomeric parameters of malignant MM cells from the peripheral blood and bone marrow were similar, suggesting that 3D telomere profiling may assess MRD in liquid biopsies of MM patients. This review focuses on the prognostic value of 3D telomere profiling in MM. 3D spatial telomere analysis may potentially address a critical unmet clinical need in managing MM and, if incorporated into current guidelines, help to accurately predict disease status, progression risk, overall survival, and response to treatment.

## 1. Introduction

Telomere dysfunction is considered one of the critical genome alterations contributing to genomic instability, which is a hallmark of cancer [1,2]. Tumor cells display an altered telomere organization that manifests in telomeric aggregates, breakage-fusion-bridge cycles, changes in gene expression, and genome destabilization [3,4]. A crucial role of genome instability and telomeric dysfunction was demonstrated in solid tumors, as well as in multiple hematological malignancies, including multiple myeloma (MM), which accounts for approximately 10% of all hematologic malignancies, with more than 35,000 new cases and 13,000 deaths reported in the United States alone each year [5]. This clonal plasma cell proliferative disorder is characterized by the abnormal increase of monoclonal immunoglobulins, leading over time to end-organ damage, such as renal dysfunction, hypercalcemia, anemia, and bone pain, accompanied by lytic lesions [6,7,8]. MM is considered an advanced stage of a whole range of monoclonal gammopathies, which progress from asymptomatic monoclonal gammopathy of undetermined significance (MGUS) to smoldering MM (SMM), and finally, to fully symptomatic MM [9].

An asymptomatic precursor stage of MM, MGUS, is present in approximately 5% of the population over the age of 50 [10]. MGUS is diagnosed based on the presence of a serum monoclonal protein (M protein) at a concentration < 3 g/dL, a bone marrow with <10 percent monoclonal plasma cells, absence of end-organ damage, such as lytic bone lesions, anemia, hypercalcemia, etc., and lack of B-cell lymphoma or other M-protein-producing disease [11]. As MGUS is clinically silent, many patients may have the condition for over a decade before detection, and treatment for MGUS is currently not recommended. However, in around 1% of MGUS patients, the disease progresses to the intermediate stage, SMM, which represents a more advanced but still asymptomatic form of MM [12]. Compared with MGUS, SMM is characterized by higher serum monoclonal protein concentrations (≥3 g/dL) in the absence of end-organ damage [13]. SMM is considered a heterogeneous disease with high variability in the risk of progression to symptomatic MM [14]. High-risk SMM carries ~50% risk of progressing to MM at 2 years from SMM diagnosis [15].

While MM is considered an incurable disease, with the average overall survival (OS) of high-risk patients of only 3–5 years, current advances in therapeutic approaches, such as quadruplet induction and immune-based therapies, have increased the median OS of low-risk MM patients to over 10 years [5,16]. However, despite novel therapies, even patients who achieve complete remission (CR) often have measurable minimal residual disease (MRD), a small population of residual malignant plasma cells [17]. This further underscores the ongoing need for precise risk stratification and subsequent development of risk-adapted treatment strategies throughout the entire spectrum of monoclonal gammopathies.

Complexity and heterogeneity are hallmarks of the MM. Over 50 identified mutated genes are significantly associated with the disease, encoding proteins involved in signaling pathways, DNA repair, transcription, cell cycle regulation, plasma cell differentiation, and cellular adhesion, among other processes [18]. Furthermore, the mutational landscape of MM changes with the progression of the disease, and after therapy [19,20]. Early stages of MM involve significant karyotypic changes in the malignant plasma cells, such as chromosomal gains or losses, translocations, and rearrangements, including translocations in the telomeric *IGH* locus (14q32.33) [21]. Telomere numbers, intensity, aggregates, and the three-dimensional arrangement of telomeres within the nucleus were identified as prognostic biomarkers in several cancers, including MM [22,23,24]. Recently, telomere-based analysis of the three-dimensional (3D) nuclear architecture of myeloma cells has gained increasing attention. Spatial telomere profiling using TeloView, a software specifically designed to quantify key 3D telomeric parameters on a single-cell level [25] has emerged as a valuable prognostic tool in MM, as it addresses a critical unmet clinical need in managing MM and can accurately predict the disease status and progression risk, overall survival, and response to treatment [26]. Furthermore, telomere profiling of cells in liquid biopsies [27] was shown to be a promising and effective method for risk-stratification and the prospective long-term monitoring of disease stability in MM patients who underwent therapy and/or bone marrow transplantation. This review will discuss telomere dysfunction, genomic instability, and their relevance in MM, with future impact on the clinic.

## 2. Telomeres and Genomic Instability

Studies of higher-order chromatin arrangements and their dynamic interactions demonstrated that chromosomes are compartmentalized into discrete territories, and such compartmentalization directly impacts the access of genes to the transcription and splicing factors [28,29]. The influence of three-dimensional (3D) nuclear chromosomal organization on gene expression and genome stability became a focus of substantial research, especially in the context of cancer. In the early stages of carcinogenesis, changes in chromosomal stability, DNA folding, and transcription are considered the main triggers of the malignant transformation [30]. Indeed, extensive chromosome rearrangements and overall genomic instability are regarded as hallmarks of cancer [31,32,33,34,35,36,37]. While these rearrangements may stem from several possible mechanisms [38], numerous studies have clearly demonstrated the crucial role of telomere dysfunction in promoting genomic instability in malignancies [39,40,41,42,43].

Telomeres cap the ends of the chromosomes, preventing end-to-end fusions, averting inappropriate DNA damage response, and protecting chromosomal ends from degradation [44]. Telomeres are composed of long tracts of double-stranded TTAGGG repeats with a single-stranded G-tail, protruding in the 3′ direction [45], and organized into a loop-like structure (T-Loop) that protects the telomere from the DNA damage response machinery [46]. This protection is enabled by the proteins of the shelterin complex, consisting of Telomeric Repeat binding Factors TRF1 and TRF2, the Repressor/Activator Protein 1 (RAP1), the bridging molecules TPP1 and TRF1 Interacting protein (TIN2), and the Protector of Telomeres 1 (POT1) [47]. The shelterin complex plays a crucial role in regulating telomere length and telomere protection, and acts as an anchoring factor for enzymes and effector proteins of various signaling cascades [48]. While somatic cells lack telomere length maintenance mechanisms, resulting in progressive shortening of the telomeric sequence with each cell cycle, in stem and germ cells, as well as in some cancer cells, telomere attrition is reversed by adding telomeric repeats de novo by the telomerase enzyme complex [49]. Indeed, telomere length changes have long been recognized as a critical mechanism in cancer pathogenesis, with a complex correlation between telomere length and the risk of various cancers [50,51,52,53,54,55]. Critical shortening of the telomeres (often transient) at the early stages of transformation has been shown to lead to a telomere crisis, a state of extensive genomic instability [56,57]. Loss of telomeric protection was shown to lead to McClintock’s breakage-fusion-bridge cycles, chromosomal abnormalities, and aneuploidy [58]. The resulting accumulation of mutations, in turn, triggers an increase in telomerase expression, which is necessary for continued cell proliferation and transition to malignancy [39]. Reactivation of the telomerase is observed in over 80% of all cancers [59], allowing the cell to avoid DNA damage signaling pathways [60]. Indeed, studies showed that activation of telomerase in cells that entered breakage-fusion cycles and have lost tumor suppressive pathways such as p53 and pRb, results in stabilization of telomeres and immortality, and, accompanied by genome instability, is a driving force of tumorigenesis [61,62,63,64].

The remaining cancers use the alternative telomere lengthening pathway (ALT) [65,66]. With the development of fluorescent in situ hybridization (FISH), a tool for visualizing genomes, ranging from the level of chromosome territories to the level of specific chromatin structures [67,68] it became apparent that telomere length is not the only marker of genomic instability during tumorigenesis. The quantification of digital FISH images (quantitative FISH or QFISH) using a fluorescent peptide nucleic acid (PNA) probe confirmed, for the first time, that telomere intensity correlates with telomere length [69]. QFISH enabled molecular cytogenetic analysis at the single-cell level, allowing the study of telomeric imbalances in interphase nuclei.

## 3. Three-Dimensional Spatial Organization of Telomeres as a New Tool for Single-Cell Analysis

Combining QFISH, which paved the way for exploring the unique features of telomeres in single cells, with spatial telomere analysis enabled moving from mere assessment of telomere length to analyzing telomere distribution in the nucleus throughout the cell cycle [22,70]. In particular, TeloView^®^, a software that utilizes the 3D analysis of six key parameters generated from the telomeres of a single cell, allowed for identification and quantification of several crucial parameters of genomic instability (Table 1), such as the number of telomeres as an indicator of aneuploidy, their length as measured by relative fluorescent intensity, number of aggregates, the spatial distribution of telomeres throughout the cell cycle (axial or *a/c* ratio), nuclear volume, and the telomere numbers to the nuclear volume ratio [22,25,69].

**Figure 1:**
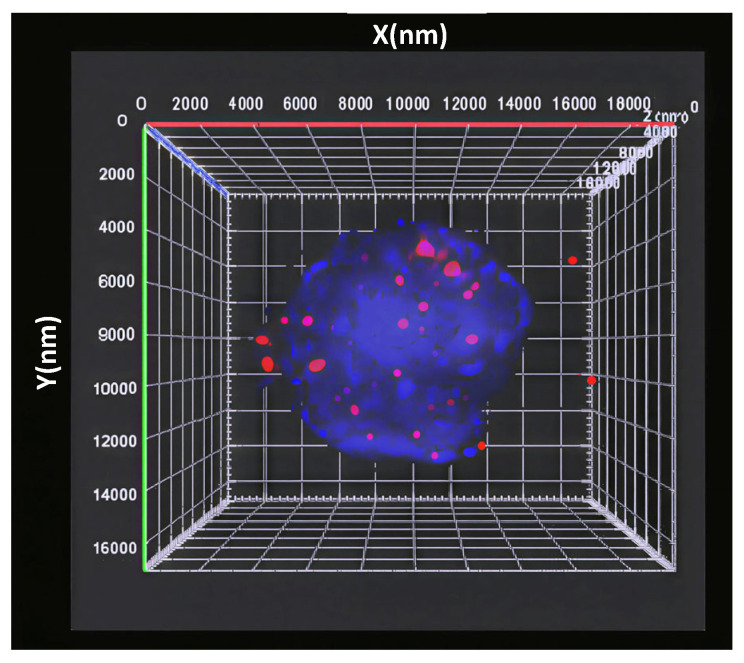
Three-dimensional distribution of the telomeres, labelled by the fluorescent probe (red), in the nucleus (DAPI, blue).

Spatial analysis revealed that the telomeres of the normal interphase nucleus exhibit a distinct 3D organization (Figure 1) that is cell-cycle-dependent [22,71]. In the G0/G1 and S phases, they demonstrate spherical-like volume distribution throughout the nucleus, and an axial (*a/c*) ratio, a measure of spatial telomeric distribution throughout the nucleus, close to 1.0. In contrast, the G2 phase of the cell cycle is associated with the 3D reorganization of telomeres into a flat telomeric disk, and a markedly higher *a/c* ratio [22].

Another characteristic feature of tumor cells is the presence of telomeric aggregates. These telomeric clusters cannot be further separated into individual telomeres at a resolution of 200 nm, and are characterized by a larger volume and stronger integrated intensity [22]. Studies showed that formation of telomeric aggregates is a multifactorial event that is linked to the deregulation of c-Myc, increased incidence of breakage-fusion-bridge cycles [70], down-regulation of the telomere repeat binding protein (TRF2) [71,72], essential for telomere capping and genome stability [73], changes in the expression of nuclear matrix and nucleoskeletal proteins [74,75] and viral infections (such as Epstein-Barr virus) [76,77,78].

Spatial telomere analysis has demonstrated its unique utility in assessing genomic instability in several solid and hematological cancers, including Hodgkin lymphoma, myelodysplastic syndromes, acute myeloid leukemia, glioblastoma, neuroblastoma, and prostate cancer [23,79,80,81,82,83,84,85,86,87]. In 2013, a proof-of-concept study by Klewes et al. [23] analyzed for the first time the 3D telomeric structure of plasma cells from patients at various stages of multiple myeloma (MM), ranging from the early precursor stage to active disease and relapsed MM. The study showed that disease progression was associated with an increased number of aggregates and t-stumps (very short telomeres) [88], accompanied by an increase in the nuclear volume of plasma cells. This was the first indication that the 3D reorganization of telomeres in myeloma cells may be potentially used for patient classification, paving the way for a growing body of research focused on the value of telomere profiling in risk-stratifying MM patients. This study is also the first to compare blood and bone marrow of MM patients and found no significant differences, thus suggesting that telomere profiling of cells in liquid biopsies can be used to assess MM status.

## 4. Risk-Stratification in MM: Current Advances and Challenges

A high heterogeneity of MM, along with its diverse genetic, molecular, and clinical profiles, drives variable disease behavior and treatment responses. Therefore, the ability to stratify patients by risk and monitor disease progression, both in the transition from precursor states to active MM, and as a measure of treatment response, plays a critical role in managing this heterogeneity.

### 4.1. Risk Assessment in Precursor States

Due to the increased risk of progression to lymphoproliferative malignancy, MGUS requires long-term follow-up, guided by risk stratification. MGUS patients are initially followed up six months after diagnosis, with subsequent visits scheduled according to the estimated risk of progression [89]. This risk is currently determined by the disease burden and the underlying cytogenetic type of the disease [90,91]. SMM is a heterogeneous disease with a significantly higher risk of progression: nearly 40% of SMM patients progress to MM within the first 5 years [26]. This makes SMM a critical stage for studying early intervention strategies and for developing sensitive risk-stratification models. Currently, risk stratification of SMM in clinical practice primarily relies on circulating plasma cell counts, the presence of M protein, serum biomarkers, and the percentage of bone marrow plasma cells (BMPC). For instance, a widely used 20/2/20 risk-stratification by Mayo Clinic classifies SMM patients into risk groups (low-, intermediate-, and high-risk) based on serum M-protein > 2 g/dL, BMPC > 20%, and an involved/uninvolved free light chain ratio > 20 [92]. These criteria were further refined by incorporating specific cytogenetic abnormalities, such as t(4;14), del(17p), hyperdiploidy with +1q, or t(14;16) [93]. Genomic profiling enabled the stratification of SMM into two categories: a more stable disease resembling MGUS and a more aggressive one, molecularly similar to MM [94]. In low-risk patients, early treatment with lenalidomide, either alone or in combination with dexamethasone, can delay progression and improve survival outcomes [95,96]. Based on the 20/2/20 criteria combined with cytogenetic assessment, a 2-year progression risk for patients with 2 or more risk factors is 44%. High-risk SMM patients are likely to progress to MM within 29 months [97].

Early risk-stratification models in MM relied solely on the disease burden [98] or the levels of serum albumin and β2-microglobulin [99]. The development of FISH technology enabled more sensitive genomics-based risk stratification, leading to the gradual integration of genomic profiling into existing clinical risk-assessment models [100,101,102,103]. In 2024, the International Myeloma Society (IMS) and IMWG incorporated chromosomal abnormalities, such as del(17p) and/or *TP53* mutation, biallelic del(1p32), and two or more intermediate-risk rearrangements, such as 1q21+, t(4;14), t(14;16), and monoallelic del(1p32), into the MM scoring model, which allowed to classify 20% of newly diagnosed MM (NDMM) patients as high-risk [104]. In addition to predicting disease progression or patient survival, risk stratification of MM is crucial for developing personalized risk-adapted therapies, both in NDMM and in relapsed MM.

### 4.2. Minimal Residual Disease

Achievement of MRD negativity emerged as a strong and independent prognostic factor in the real-world setting [105]. While MRD assessment has become a validated endpoint in clinical trials [106], its impact on treatment decisions is evolving. The current International Myeloma Working Group consensus criteria for response and MRD assessment require quantifying tumor burden after therapy in the bone marrow using highly sensitive methods, such as next-generation sequencing, next-generation flow cytometry, and imaging-based techniques (PET/CT, MRI) [107]. However, evaluating tumor burden alone cannot account for the inherent heterogeneous nature of MM, nor can it assess the development of treatment-resistant clones. It also fails to address clinically relevant questions that are essential for establishing a standard of care, such as whether it is safe to discontinue treatment with sustained MRD or whether MRD positivity justifies a new treatment. In NDMM patients who are candidates for autologous stem cell transplantation (ASCT), induction treatment combines the anti-CD38 monoclonal antibody daratumumab (D) with the triplet regimen of bortezomib, thalidomide, and dexamethasone (VTd) or bortezomib, lenalidomide, and dexamethasone (VRd), followed by transplantation [108]. However, in patients who are not high-risk, the ASCT may be delayed until the first relapse. Furthermore, while standard-risk patients may benefit from lenalidomide maintenance, some studies show that high-risk MM patients may require a bortezomib plus lenalidomide maintenance regimen [109]. At relapse, a triplet regimen is usually needed, and the choice of regimen varies with each successive relapse. In cases of heavily relapsed/refractory (R/R) patients, chimeric antigen receptor (CAR) T-cell and T-cell redirecting bispecific antibody (BsAb) therapies have been shown to significantly improve response rates and durations [110]. In this setting, assessing treatment response and detecting high-risk MRD become crucial.

Clinically recognized MRD detection approaches, such as next-generation sequencing (NGS), mass spectrometry (MS), and multiparametric next-generation flow cytometry (NGF) are limited to mere quantification of plasma cell sequences or plasma cells, respectively, in the bone marrow sample, without providing information on genomic instability present in single myeloma cells [111,112]. This further emphasizes that currently used risk-stratification approaches fail to identify the diversity of individual tumor cells and cannot distinguish between biologically different subclones associated with aggressive or indolent behavior. Given the heterogeneous nature of MM, developing single-cell–based approaches may enable more accurate identification of high-risk cellular populations and truly personalized disease monitoring.

## 5. Telomere Maintenance in Multiple Myeloma: Beyond Telomeric Length

Numerous studies demonstrated that the risk of developing MM is influenced by genetic variability [113,114,115,116,117,118,119]. Karyotypic changes, including chromosomal gains or losses, translocations, and complex genomic rearrangements in malignant plasma cells, are considered a classical hallmark and an essential prognostic factor of MM [120,121,122,123,124]. Of them, translocations involving the immunoglobulin heavy chain region at chromosome 14q32 are most common in B-cell malignancies and are observed in approximately 40% of patients with MM [125]. The locus is positioned at the distal end of chromosome 14, within the telomeric region. Notably, several partner loci that participate in recurrent translocations (such as 4p16, 6p25, and 16q23) are also positioned at telomeric or subtelomeric regions, further confirming the potential role of spatial telomeric and chromosomal organization in these chromosomal alterations [18].

### 5.1. Telomere Length in MM

Mounting evidence links MM risk to defects in telomere biology. Alterations in telomere length and architecture have been documented in plasma cell disorders [126]. In MM, malignant plasma cells exhibit increased telomerase activity and sustain short but stable telomeres [127,128,129]. A 2012 study by la Guardia et al. [130] used conventional methods of telomere assessment, such as cytogenetic analysis, telomere FISH, and spectral karyotyping (SKY), to show a high incidence of chromosomal translocations in chromosomes with loss of telomeric signals as a result of telomere shortening in MGUS and MM patients. Furthermore, telomeric attrition inversely correlated with the increase in the percentage of telomeric aggregates [130]. Hyatt et al. [131] employed Single Telomere Length Analysis (STELA), a high-resolution technology for detecting telomeres within the telomeric fusion length ranges, to generate telomere length profiles of MGUS MM patients. The study revealed that both MGUS and MM bone marrow cohorts exhibited considerable heterogeneity in telomere length [131]. Similarly, Wu et al. [127] evaluated the telomere length in bone marrow of 115 MM patients by TRF Southern blot analysis to report a significant positive correlation of telomerase activity and a negative correlation of telomere length with poor prognosis features of MM, suggesting that telomeric length is a prognostic marker in MM [121,131]. However, a specific subset of MM patients present with unusually long telomeres, related to the Alternative Lengthening of Telomeres (ALT) pathway during disease progression [127,132,133,134,135]. This phenomenon of ALT involves inter-telomeric homologous recombination, reported primarily in carcinoma-derived cell lines [136], and further emphasizes the high inherent heterogeneous genetic landscape of MM.

### 5.2. Spatial Telomere Organization in MM

The development of more advanced QFISH technology led to a more accurate assessment of the clonal diversity inherent to MM. Single-cell analysis using QFISH enabled the early detection of treatment-resistant, metastatic clones, indicative of clonal evolution and aggressiveness [137]. When Klewes et al. [23] looked at the single-cell 3D telomere profiles of blood and bone marrow samples of patients diagnosed with MGUS and MM, as well as of patients who went into relapse (MMrel), they were able to show that during disease progression from MGUS to MM and MMrel, the number of aggregates and t-stumps, and the nuclear volume of plasma cells increased. Furthermore, disease progression from MGUS to MM to MMrel was associated with a gradual increase in telomere attrition. The average number of telomeric aggregates was also increased in myeloma cells that survived treatment (MMrel), thus suggesting an association with relapsed myeloma. However, the difference in the number of aggregates between MM and MMrel was not significant, which implies clone expansion of malignant plasma cell survivors or a constant level of dynamic 3D telomere remodeling without further increase in aggregates. This was the first study to demonstrate the utility of telomere profiles and 3D nuclear architecture of MM cells in patient stratification, considering the high heterogeneity of MM. The study was also the first to describe automated analysis of telomeric features to detect minimal residual disease (MRD). This study paved the way to a plethora of subsequent studies of spatial telomere organization in the context of MM.

A 2019 study by Yu et al. [138] analyzed blood and bone marrow samples from 37 patients diagnosed with MGUS and 25 newly diagnosed with MM, and demonstrated a significant decrease in the average signal intensity (indicative of shorter telomere lengths), substantially shorter telomeres, an absence of very long telomeres, and an increased number of telomeres in MM cells compared to MGUS. MM plasma cells also had somewhat lower *a/c* ratio. A 2021 study by Rangel-Pozzo et al. [82] compared different 3D telomere parameters from bone marrow samples of 214 untreated patients (54 MGUS, 24 SMM, and 136 MM) with a clinical follow-up of at least 60 months to investigate a potential prognostic role of telomere assessment in the spectrum of myeloma at diagnosis. The results demonstrated that the number of telomeres, their intensity, the number of aggregates, and the *a/c* ratio differed significantly across the MM spectrum. Moreover, the degree of genomic instability correlated with the disease stage, with the highest combined telomere changes found in the SMM and MM subgroups. Stratifying SMM patients based on 5-year clinical follow-up data (progression and survival) revealed that telomere profiles were a sensitive marker of disease aggressiveness, identifying two distinct groups of SMM patients: biologically inactive MGUS-like and malignant MM-like. SMM patients in the second group had higher telomere numbers and intensity, more telomeric aggregates, a higher *a/c* ratio, and a greater nuclear volume.

The study also showed that MM patients with progressive disease exhibited increased telomere signals, a higher number of telomere aggregates, and a higher *a/c* ratio, along with a decrease in nuclear volume, compared to MM patients with stable disease.

Furthermore, total and average telomere intensities were associated with shorter OS in the SMM and MM groups, confirming that telomere-related genomic instability correlates with disease aggressiveness and can assist in identifying high-risk patient populations.

Notably, in MM, the average intensity (telomere length below 13,500 arbitrary units) and an increased number of telomere aggregates (≥3) emerged as prognostic factors to identify high-risk patients with the progressive disease. This study [82] provided the first evidence that prediction modes based on the single-cell analysis of telomeric parameters may be a potentially valuable tool if incorporated into existing risk stratification guidelines in clinical practice.

## 6. 3D Telomere Profile-Based Prediction Models for MM

Quantitative telomere analysis demonstrated that in both SMM and MM patients, differential telomere parameters enable the identification of stable or progressive disease and stratification of patients into high-risk and low-risk groups, and paved the way to incorporating telomere profiling into clinical scoring models. Such risk prediction models, developed based on telomere parameters and the 3D architecture of the nucleus, can be potentially used to guide evidence-based treatment decisions for MM patients at every stage of the disease progression, from MGUS to SMM to MM. Furthermore, telomere profiling may potentially predict the risk of relapse in MM patients post-treatment.

A proof-of-concept prospective study of 21 SMM patients revealed notable differences in telomeric parameters between SMM patients who progressed to MM within 2 years and those who remained stable for over 5 years [139]. The disease progression of high-risk SMM patients was confirmed clinically by MM-caused morbidity. SMM patient groups demonstrated distinct telomere profiles in the stable patients compared to those who progressed to full-stage MM across five independent telomeric parameters. A follow-up clinical validation study used an expanded cohort of 168 SMM patients, with 53 patients who progressed to symptomatic MM within 24 months (i.e., short progression) and 35 patients who remained in the SMM stage for 5+ years (i.e., long progression) [26]. Telomere length, the *a/c* ratio, the distribution of telomeres in the spatial volume of the nucleus, and nuclear volume were identified as predictors suitable for regression modeling. A developed predictive scoring model was able to stratify individual SMM patients based on the risk of progression to active MM. The highest sensitivity and specificity were achieved using 3 predictors, including the total telomere length, *a/c* ratio, and distribution of telomeres in the nuclear space. The predictive scoring model of the training set demonstrated superior accuracy of 80%, with sensitivities and specificities of 0.75 and 0.70, respectively. Furthermore, blind validation of the model using an additional cohort of 41 patients with short progression and 25 patients with long progression achieved a positive predictive value of 85% and a negative predictive value of 73%, with corresponding sensitivity and specificity of 83% and 76%, respectively. For the first time, a scoring model based on 3D telomere profiling was used as an accurate prognostic tool to stratify SMM patients into their respective risk groups. This scoring model, thus, may potentially contribute to evidence-based decision-making regarding treatment approaches for “high-risk” SMM patients and enable confident monitoring of “low-risk” SMM patients without therapeutic intervention.

Identifying NDMM patients with a high risk of developing drug resistance remains an important clinical need. Therefore, identifying newly diagnosed MM (NDMM) patients who will develop resistance to treatment before relapse may allow switching these patients to another treatment regimen to potentially prevent the relapse. A 2023 study [140] of 178 NDMM patients who were followed longitudinally for up to 5 years, identified several MM risk groups, suggesting a clinical relevance of telomere profiling in predicting the risk of developing drug resistance and relapse up to ~13 months before the relapse itself.

The developed predictive scoring model that included telomeric parameters, such as telomere length, number of detectable telomeres, % of telomere stumps and the *a/c* ratio was able to predict the risk of relapse at the level of the individual patient with a specificity of 85% and sensitivity of 72%. Spatial telomere profiling, thus, emerged as a practical and accurate prognostic biomarker of the risk of NDMM patients to develop resistance to first-line therapy combinations.

The demonstrated utility of telomeric profiling in risk-stratification of MM and SMM strongly suggests that developing a similar scoring model for post-treatment patients with MRD may allow us to move beyond merely detecting and enumerating plasma cells, which are currently used in clinical practice. Telomere characteristics of residual MM clones may enable the identification of MRD positivity or negativity, concomitant with profiling the level of aggressiveness of the cells and clones identified. Furthermore, current technologies, such as NSG, flow cytometry, and mass spectroscopy, are predominantly applicable to bone marrow specimens [17,141]. This limits their applicability to monitor patients continuously, and does not take into account the heterogeneity of the MM. These limitations may be overcome by blood-based MRD evaluation. Levels of CTCs in the peripheral blood of myeloma patients are increasingly recognized as a prognostic biomarker for risk stratification in MM [142] and identified as an independent prognostic factor in the context of the most effective standard of care for transplant-eligible NDMM [142,143]. A recent proof-of-concept study demonstrated concordance between 3D telomere profiles of myeloma plasma cells isolated from marrow samples or circulating myeloma plasma cells isolated from peripheral blood (liquid biopsy) of transplant-eligible MM patients [27]. These results demonstrated the potential of the 3D telomere profiling to monitor MRD in MM patients based on peripheral blood samples, liquid biopsies, from the point of diagnosis, without the need for marrow samples, potentially presenting a new generation of a minimally invasive assessment of disease stability or progression that will rely on assessing genomic instability for risk stratification beyond simple enumeration.

## 7. Incorporating Telomere Profiling into Current MM Risk-Stratification Frameworks: Future Perspectives

In light of the demonstrated value of 3D telomere profiling as a prognostic biomarker in MM, combining telomere profiling with currently accepted MM and SMM risk-stratification guidelines may offer a major advancement toward biologically informed, precision medicine. Telomere architecture on a single-cell level captures cellular heterogeneity that conventional models overlook. Therefore, integrating 3D telomere biomarkers with established clinical parameters may allow for better identification of high-risk patients likely to progress to symptomatic MM, refine monitoring strategies and treatment timing, bridge molecular and clinical risk assessment, and enhance early detection of aggressive disease.

The 2024 study by Kumar et al. [26] assessed the correlation between the newly developed SMM scoring model based on 3D telomere profiling and the Mayo 20-2-20 scoring model currently used in clinical practice. The SMM cohort of 110 patients included 71 patients who were scored as high and 39 as low risk by the 20-2-20 model. The overall concordance between the telomere-based prediction and the 20-2-20 score was 53% (58/160) for the entire cohort, and 49% and 59% for short progression and long progression cohorts, respectively.

In a continuing analysis, 88 SMM patients from the original modeling cohort (low/intermediate and high risk of progression) were used to combine the SMM model. Developed based on the telomere profiling (which included parameters, such as a/c ratio, the total number of telomeric signals, and average distance of telomeres to the nuclear center) with the risk assessment of 2/20/20. The combination was able to identify high-risk SMM patients better than each scoring model alone. The reported receiver operator characteristics (ROC) for the 2/20/20 and the telomeric SMM scoring models were 0.64 and 0.77, respectively, whereas the ROC for the combined scoring was 0.80 [26]. The results clearly demonstrated that combining both scoring methods may considerably improve risk assessment and strongly suggest that telomere profiling has clinical value for future inclusion in SMM guidelines.

## 8. Conclusions

Three-dimensional telomere profiling represents a promising prognostic biomarker in MM, addressing a critical unmet need for more precise disease assessment. Quantitative telomere profiling evaluates spatial and structural dynamics of telomeres at the single-cell level, provides valuable insights into genomic instability and clonal evolution of MM, enables prediction of disease stage, progression risk, overall survival, and treatment response, and is potentially valuable for evidence-based treatment decisions. Including 3D telomere profiling in currently accepted models of MM risk stratification could enhance prognostic precision and support the development of personalized, risk-adapted therapeutic strategies.

## Figures and Tables

**Table 1:** Telomeric parameters and their biological significance.

Parameter	Biological Significance	
Telomere length (signal intensity)	Quantitative measure of fluorescence intensity of telomere signals.	Reflects average telomere length; shorter telomeres indicate genomic instability.
Number of telomere signals per nucleus	Counts discrete telomere signals detected in each nucleus.	Indicates chromosome number integrity and overall nuclear organization.
Number of telomere aggregates (clusters)	Measures the presence of closely associated or fused telomere signals.	High aggregation suggests telomere dysfunction and chromosomal instability.
Nuclear volume	Three-dimensional measurement of nuclear size.	Correlates with cellular state and malignancy progression.
*a/c* ratio	Ratio of the short (a) to long (c) nuclear axis.	Reflects cell-cycle progression; low values indicate spherical nuclei typical of proliferating cells.
Spatial distribution of telomeres	Quantifies the positional organization of telomeres within the nucleus.	Altered spatial patterns are associated with genomic instability and disease progression.

## Data Availability

No new data were created or analyzed in this study.
